# PCR Master Mixes Harbour Murine DNA Sequences. Caveat Emptor!

**DOI:** 10.1371/journal.pone.0019953

**Published:** 2011-05-25

**Authors:** Philip W. Tuke, Kate I. Tettmar, Asif Tamuri, Jonathan P. Stoye, Richard S. Tedder

**Affiliations:** 1 Transfusion Microbiology Research and Development, National Transfusion Microbiology Laboratories, National Health Service Blood and Transplant, Colindale, London, United Kingdom; 2 Blood Borne Virus Unit, Virus Reference Department, Centre for Infections, Health Protection Agency, Colindale, London, United Kingdom; 3 Division of Mathematical Biology, Medical Research Council National Institute for Medical Research, Mill Hill, London, United Kingdom; 4 Division of Virology, Medical Research Council National Institute for Medical Research, Mill Hill, London, United Kingdom; Columbia University, United States of America

## Abstract

**Background:**

XMRV is the most recently described retrovirus to be found in Man, firstly in patients with prostate cancer (PC) and secondly in 67% of patients with chronic fatigue syndrome (CFS) and 3.7% of controls. Both disease associations remain contentious. Indeed, a recent publication has concluded that “XMRV is unlikely to be a human pathogen”. Subsequently related but different polytropic MLV (pMLV) sequences were also reported from the blood of 86.5% of patients with CFS. and 6.8% of controls. Consequently we decided to investigate blood donors for evidence of XMRV/pMLV.

**Methodology/Principal Findings:**

Testing of cDNA prepared from the whole blood of 80 random blood donors, generated gag PCR signals from two samples (7C and 9C). These had previously tested negative for XMRV by two other PCR based techniques. To test whether the PCR mix was the source of these sequences 88 replicates of water were amplified using Invitrogen Platinum Taq (IPT) and Applied Biosystems Taq Gold LD (ABTG). Four gag sequences (2D, 3F, 7H, 12C) were generated with the IPT, a further sequence (12D) by ABTG re-amplification of an IPT first round product. Sequence comparisons revealed remarkable similarities between these sequences, endogeous MLVs and the pMLV sequences reported in patients with CFS.

**Conclusions/Significance:**

Methodologies for the detection of viruses highly homologous to endogenous murine viruses require special caution as the very reagents used in the detection process can be a source of contamination and at a level where it is not immediately apparent. It is suggested that such contamination is likely to explain the apparent presence of pMLV in CFS.

## Introduction

A novel retrovirus, xenotropic murine leukaemia virus-related virus (XMRV), probably of mouse origin, was identified in nucleic acid (NA) extracted from human prostate cancers using a multi-virus array [1]. Initial US studies indicated a high prevalence of infection in patients with prostate cancer (PC) suggesting a possible aetiologic link [1], although this remains unproven. Intravenous inoculation of macaques with XMRV caused infection with a short plasma and cellular viraemia followed by subsequent seroconversion [2].

Late in 2009 Lombardi and colleagues reported the presence of XMRV DNA in peripheral blood leucocytes from 67% of patients with chronic fatigue syndrome (CFS) and 3.7% of healthy controls [3]. They subsequently amended the prevalence to 7% as measured by PCR of genomic DNA and stated that cDNA synthesis was required to achieve the 67% prevalence [4]. In addition the original study reported the isolation of XMRV from patients with CFS from both peripheral blood leucocytes and from plasma. Lo and colleagues using predominantly archival material from patients with CFS detected a high prevalence (86.5%) of pMLVs similar to, but different from, XMRV [5]. Other studies failed to demonstrate a link between XMRV infections and CFS [6], [7], [8], [9], [10]. Studies of XMRV infection in patients with PC indicate that XMRV is rarely present in prostatic cancer tissues and that antibody to XMRV cannot be detected reliably in the plasma of patients with PC [1], [11], [12], [13], [14], [15]. It has been suggested that the failure to detect XMRV NA in such patients is due to the use of DNA detection protocols rather than those targeting RNA [4], [16]

While developing a sensitive and robust detection protocol for viral RNA in blood samples we encountered what appeared to be contamination of PCR reagents. We examined one potential source of contaminating murine retroviral sequences using protocols derived from Urisman [1], Lombardi [3] and Lo [5] and their colleagues.

## Materials and Methods

Whole blood samples were obtained from Donation Testing at the National Health Service Blood and Transplant (NHSBT) centre at Colindale, London NW9. Nucleic acid was extracted from 273 µl of whole blood on a Qiagen MDx Bio-robot and eluted into 80 µl of AVE buffer. Nuclease-free water (Severn Biotech) was used throughout for the cDNA and PCR mix preparations and as no template controls. cDNA synthesis was undertaken on 80 NA extracts that did not contain detectable gag XMRV proviral sequences as determined using a previously described TaqMan assay [17] but performed using reagents as detailed in Table 1. A total volume of 20 µl contained 1× IPT PCR buffer, 0.5 mM each dNTP, 5 mM MgCl_2_, 6.7 ng/µl random hexamers (Invitrogen), 6.8 U RNAsin (Promega), 100 U MMLV RT (Invitrogen). Synthesis conditions were 37°C for 90 mins, 95°C for 5 mins.

**Table 1:** Details of PCRs performed.

PCR	Primers and probes	Reagents	Conditions
gag R1gag R2	419F 1154R [3]gagIF gagIR [1]	Invitrogen Platinum Taq	4 min at 94°C(1 min 94°C, 1 min 57°C, 1 min 72°C)×45 cycles and 10 min 72°C.
gag R1gag R2	419F 1154R [3]gagIF gagIR [1]	Applied Biosystems Taq Gold LD	10 min at 95°C(1 min 95°C, 1 min 57°C, 1 min 72°C)×45 cycles and 10 min 72°C.
XMRV Taq Man	XMRV Probe, F, R [17]	Qiagen Quanti Tect Probe kit	15 min at 95°C(15 secs 95°C, 1 min 60°C)×60 cycles.
XMRV/pMLV Taq Man	P2, F3, R4	Qiagen Quanti Tect Probe kit	15 min at 95°C(15 secs 95°C, 1 min 60°C)×60 cycles.
XMRV/pMLV with IAPE Taq Man	P2, F3, R4IAPE D1Probe, D1F, D1R	Invitrogen Platinum Taq	4 min at 95°C(15 secs 95°C, 1 min 72°C)×60 cycles.
CDC pol	XPOLOF XPOLOR [20] XPOLIF XPOLIR [20]	Invitrogen Platinum Taq	4 min at 94°C(30 secs 94°C, 30 secs 57°C, 1 min 72°C)×45 cycles and 10 min 72°C.

400 nM concentrations of primers and 200 nM probes were used in all the TaqMan assays.

A modified XMRV *gag* TaqMan assay, using probe P2 and primers F3 and R4, redesigned to co-detect the pMLVs described by Lo and colleagues, was also used to analyse the cDNAs. The assay conditions were as previously described [17] however, the Qiagen Quantitect PCR probe mix was used. Details of this and subsequent PCR reactions are listed in Table 1.

P2 5′-Fam-CCG ACA GCT CCC GTC CTC CCG-Tamra-3′
F3 5′-ACC GTT TGT CTC TCC TAA AC-3′
R4 5′-AGG GTA AAG GGC AGA TCG-3′

To detect murine genomic DNA sequences a TaqMan assay to the LTR sequence of a reconstructed ancestral intra-cisternal A type particle (IAP) sequence [18] was designed using Beacon Designer 7.9 software (Premier Biosoft). It was able to detect 0.01 pg of Balb/c DNA. The probe and primer sequences were:

IAPE D1 Probe 5′-Cy5-CGT CCG TCT GAT CGT GCT TC BHQ3-3′
IAPE D1 F 5′-CTG TGT TAA CCC TAT TTC TC-3′
IAPE D1 R 5′-GAG AGG CTT AAA TAT GAG AC-3′

IAP LTR sequences were sought at the same time as XMRV/pMLV in the IPT reagents by TaqMan PCR under the following conditions: Total volume of 25 µl containing 1× IPT PCR buffer, 0.2 mM each dNTP, 2.5 mM MgCl_2_, 0.625 U of Platinum Taq DNA Polymerase (Invitrogen) and ROX was added to the PCR master mix as specified by the manufacturer of the QPCR machine.

PCR products were purified and sequenced. Sequences 1F, 1F_131010, 2D, 3F, 7C, 7H, 9C, 12C and 12D are deposited with GenBank, with the following respective GenBank accession numbers JF491136, JF491128, JF491134, JF491135, JF491129, JF491132, JF491130, JF491131 and JF491133. Sequence homology searches were performed using the EBI server and FASTA programme. To examine the relationship between our sequences and other MLVs, we aligned them to a collection of endogenous MLVs [19], XMRV VP62 [1], the sequences described by Lo and colleagues [5] and subsequent database submission (HQ601957–HQ601962) using MegAlign (DNAstar) or MUSCLE [X]. RAxML [Y] (GTR model [Z], Gamma-distributed rates) was used to create the tree topology and optimise the branch lengths. Bootstrap values were estimated from 200 replicates. Branches with less than 50% support were collapsed.

## Results

**Figure 1:**
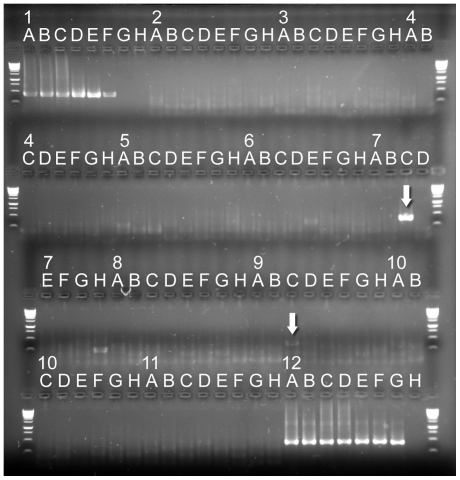
Agarose gel analysis of R2 from experiment 1. *gag* nested PCR for 80 cDNAs derived from blood donors and controls. Lanes 1A–1H ten fold dilution series of XMRV TCS (Lane 1A equivalent to 5.5×10^6^ to 5.5×10^−1^ cDNA molecules/PCR – determined by Poisson distribution). Lanes 2A–11H 80 cDNAs derived from blood donor whole blood. Lanes 12A–12H ten fold dilution series of Balb/c DNA 10^5^ pg to 0.10 pg, negative.

In initial experiments designed to test methodology for screening the blood supply, we examined 80 cDNAs from normal blood donors using the modified XMRV *gag* TaqMan protocol. None contained detectable XMRV/pMLV sequences. Subsequently, in experiment 1 (Table 2) *gag* amplification using IPT was performed on these same 80 cDNAs, essentially as previously described by Urisman [1], Lombardi [3] and Lo [5]. Two samples, 7C and 9C, gave visible bands of apparently the anticipated size (413 bp) in round two (R2) (Fig. 1). 7C also gave a visible band of the anticipated size (c 730 bp), in round one (R1). When the IPT reagents were substituted with ABTG reagents in a second set of experiments, re-amplification of 8 cDNAs, including 7C and 9C, using the nested *gag* primers failed to generate any detectable products. Using the pol nested PCR primers described by Switzer and colleagues [20] (Table 1) to re-amplify 8 cDNAs, including 7C and 9C, the cDNA of 9C failed to amplify and only 7C amplified a visible product in R2 (Experiment 3, Table 2).

**Table 2:** Details of sequences obtained.

Experiment/Source material	Sequence Identity/and size	Class	gag Homology % to XMRV VP62_DQ399707	gag Homology % to CFS1 (pMLV M630562)	Most homologous	Homology %	Source M. musculus unless stated
1/WB cDNA	7C, 372 bp	Modified polytropic	(96.8%)	(98.4%)	AC153369	99.7/372 bp	10 BAC RP23-103E4
1/WB cDNA	9C, 311 bp	Xenotropic	307/374 (82.1%)	300/374 (80.2%)	FB579941	98.3/176 bp	XMRV Patent WO2006110589
2/Water	2D, 373 bp	Polytropic	360/373 (96.5%)	365/373 (97.9%)	AC184103.1	99.7/373 bp	chromosome Y M. spretus BAC clone CH35-73N5
2/Water	3F,352 bp	Xenotropic-	345/373 (92.5%)	335/373 (89.8%)	M26006	100/352 bp	endogenous retrovirus truncated gag gene, clone del env-2 15.3
2/Water	7H, 373 bp	Polytropic	358/373 (96.0%)	363/373 (97.3%)	AC093923.3	100/373 bp	RP23-174H21
2/Water	12C, 373 bp	Modified polytropic	362/373 (97.1%)	368/373 (98.7%)	AC127319.4	100/373 bp	chromosome 5 BAC clone RP23-229N3
2/Water	12D, 373 bp	Polytropic	358/373 (96.0%)	363/373 (97.3%)	CR954969.10	100/373 bp	chromosome X clone RP23-332J14
3/WB cDNA	7Cpol, 168 bp	N/A	N/A	N/A	AC117614.14	100/168 bp	chromosome 5, clone RP23-110C17

WB = Whole Blood.

7C was a modified polytropic murine retrovirus (mpMLV) gag sequence, related to (96.8% homology), but distinct from XMRV. 7C was most closely related (with 99.7% homology) to an endogenous MLV (AC153369 10 BAC RP23-103E4). 7C was essentially identical (99.7%) to 12C (obtained subsequently from water, see below) differing by only one nucleotide in the 373 bases. 9C was a xenotropic virus sequence most closely related to XMRV (98.7% homology) but with a 45-base pair deletion (Table 2). The *pol* sequence from 7C had 100% homology with a MLV sequence AC117614 (Table 2) and was highly similar to AF033811 Mo-MLV (98.2% homology).

We next tested 88 replicates of no template (water) controls using the nested *gag* primers and two enzyme systems (Experiment 2, Table 1). All 88 replicates of water control wells were unreactive when amplified by ABTG. IPT produced four R2 visible products (2D, 3F, 7H and 12C Fig. 1). 12 C was also visible in R1. Sequencing confirmed high identity to a number of *gag* loci in the mouse genome (Table 2). Re-amplification of the four R1 products which generated a R2 signal and four adjacent “water controls” but using ABTG and *gag* IF IR only re-amplified the R1 positive sample 12C with a sequence identical to that found previously. One of four “water controls” (12D), previously unreactive gave an amplicon 12 D which was identical to a murine endogenous *gag* sequence CR954969 (Table 2).

Hence five different murine related retroviral sequences were amplified in R2 from 88 waters but only where the R1 had been generated with IPT. The further investigation of the R1 products makes it most likely that excipient material within the Invitrogen enzyme and master mix was the source of these sequences. In total seven discrete MLV sequences were amplified, two in the cDNA amplifications (Experiment 1) and five in the amplifications of the water (Experiment 2). The water itself is not the source of these sequences as all 88 were negative in the nested gag ABTG amplifications. The sensitivity of the two amplification methods were shown to be equivalent both on XMRV derived cDNA and Balb/c DNA (data not shown).

The TaqMan Q-PCR set up using the IPT PCR reagents multiplexed with the XMRV/pMLV with IAPE-D1 components, generated 19 signals (21.6%) from 88 water negative controls. The XMRV/pMLV amplifications produced 15 signals (17%) and the IAPE-D1 Q-PCR 4 (4.5%). No analyte gave both amplifications. The Ct's were late for all these reactions, between 37.8 and 46.0 for the XMRV/pMLV and 37.8 and 39.3 with the IAP-D1 Q-PCR; equivalent to 0.1 pg or less of murine DNA per reaction. These 19 amplifications each occurred independently, there were no co-amplification dual positives, these frequencies are consistent with the presence of a single target molecule per reaction.

**Figure 2:**
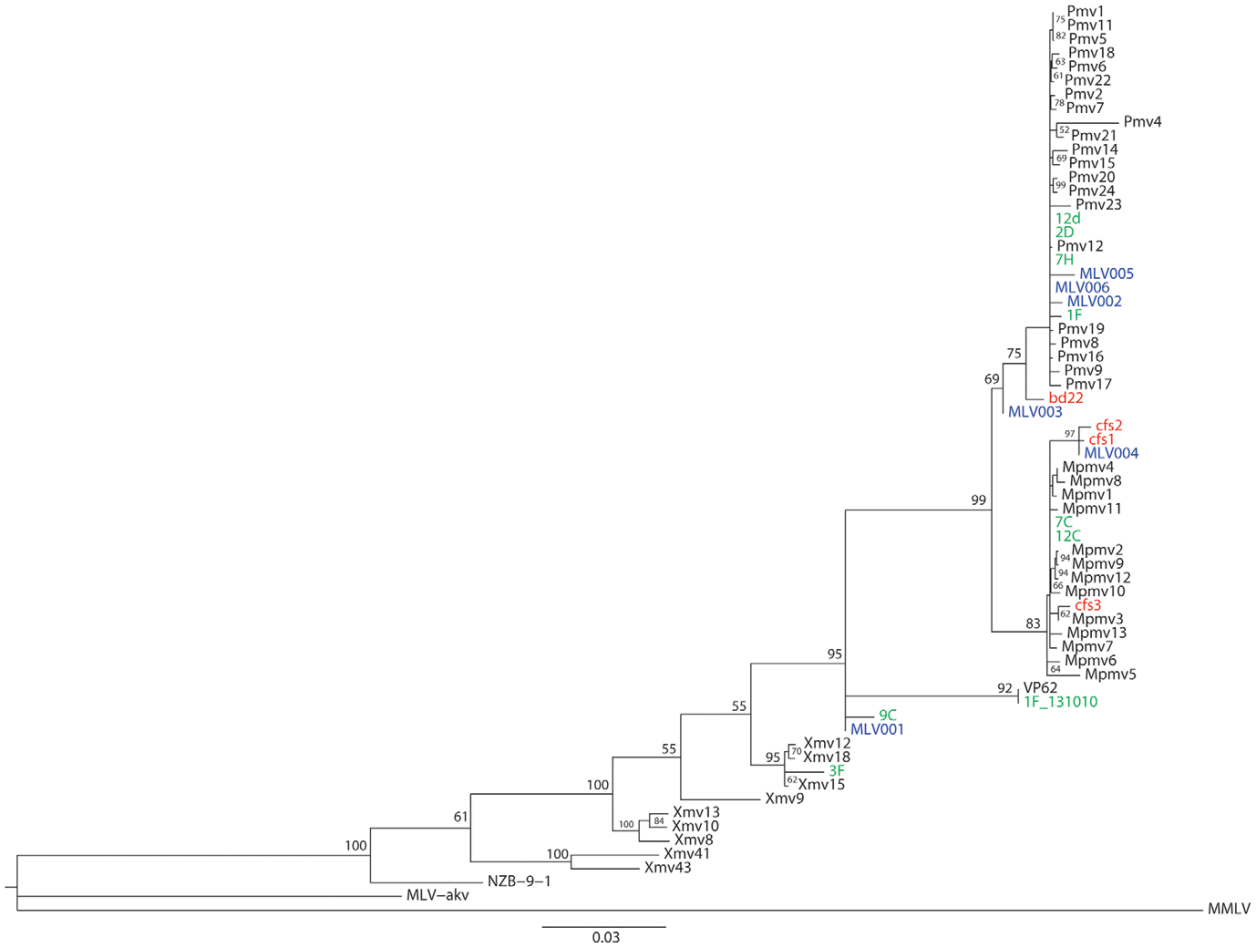
Maximum likelihood tree of gag sequences. The MLV RAxML tree for gag sequences lying between XMRV primers gagIF and gagIR was developed from the endogenous MLV sequences identified by Jern et al [19], sequences derived from CFS patients by Lo et al [5] (shown in red and blue) and the sequences amplified in this study (in green). In addition to sequences from the RNA and DNA, “1F_131010” and “1F” are included. 1F_131010 was derived from the amplification of cDNA synthesized from RNA extracted from the tissue culture fluid supernatant of an XMRV culture, kindly provided by Professor M McClure. 1F was derived from the amplification of Balb/c mouse DNA (Sigma). Bootstrap values >50 are indicated.

The results of the sequence alignments are shown as a phylogenetic tree (Figure 2) and confirm those obtained in FASTA searches. Sequences 12D, 2D and 7H fall within the pMLVs whereas 7C and 12C are mpMLVs. 3F and 9C cluster with xenotropic MLVs and XMRV. Interestingly 9C carried the same deletion as MLV001 (HQ601957), which was amplified from a patient with CFS.

## Discussion

The *gag* sequences described (Table 2) should be detectable by our modified Taq Man assay with the possible exception of one, a xenotropic sequence (3F) that carried a T to A change four bases in from the 3′ end of the forward primer. The confirmed existence of murine sequences in the IPT enzyme underline real concerns over the source of similar reported sequences. The higher detection rate in the TaqMan assay, 15 positives against the initial finding of 4 by nested PCR is what one would expect as the TaqMan sequence amplified is smaller. If these “positives” were sample derived the absence of a signal from the murine detector channel (IAP) would have led them to be mistakenly considered real. However, our interpretation is that the lack of concordant positives is because very low levels of DNA were present, that it is probably very fragmented and that small volumes will not necessarily contain both targets. These findings suggest that probing for murine genomic DNA, for example using IAP PCR, although advisable to identify overt murine DNA contamination, may not always be sufficient to exclude very low copy contaminant sequences.

XMRV research is a contentious field with controversy over both the discrepant findings of different groups and the reasons behind this. Contamination has been suggested as a problem, from sequences contained in positive samples, amplified products and plasmids. To this, it is now appropriate to add the presence of murine material either in the general laboratory environment, in reagents used in extraction and processing and now in the reagents used to detect XMRV/pMLV sequences. Robinson and colleagues have just demonstrated that murine sequences can be present in prostate sections and give false positives when searching for XMRV [21]. A study of XMRV in patients with CFS or chronic immunomodulatory conditions, using IPT reported a gag sequence with >99% homology to a mouse endogenous retrovirus [22]. This was designated as contamination although the source of this sequence was not speculated. Sato and colleagues recently reported finding sequences, predominantly RNA, related to a pMLV in IPT containing reagents [23]. Another study just published, concluded that the detection of MLV-related sequences in human samples could be due to contamination with mouse DNA, most likely contained in various laboratory reagents [24]. Finally, a phylogenetic overview concluded that the proviral sequences present in the genome of 22Rv1 cell line were ancestral to the published XMRV sequences [25].

Our data was similar to that of Oakes and colleagues in that a variety of MLV sequences were detected, however, our results were not related to the sample input which in our case were water replicates. Similarly, Sato and colleagues had found sequences derived from Invitrogen RT-PCR reagents, however they found a single pMLV rather than a variety of MLV sequences as Oakes and ourselves describe.

**Figure 3:**
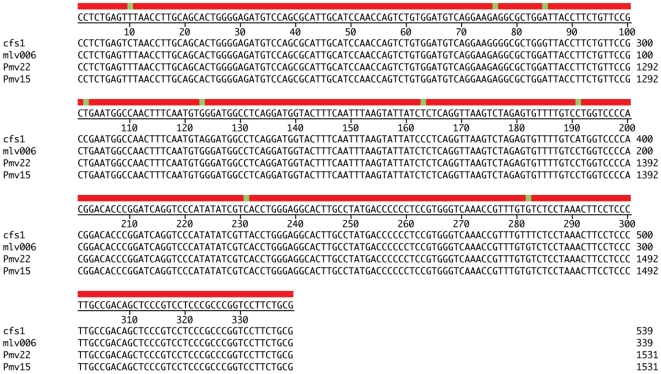
Alignment between cfs1, MLV006, Pmv15 and Pmv22. The nine nucleotide differences between cfs1 and the other viral sequences are highlighted.

The exact variety and nature of our sequences show very close parallels to those reported by Lo and colleagues from patients with CFS [5] especially in the set of samples from recent repeat isolations. They argue that the recent sequences (MLV001–MLV006; HQ601957–62) show evidence of viral evolution from an earlier sequence (assumed here to be cfs1 since this was identified in 18/21 sequences). However such evolution would be predicted to show monophylogeny. Our maximum likelihood analysis of these sequences is clearly inconsistent with such a prediction (Figure 2). In particular we see no obvious explanation for a sequence of the modified polytropic cfs1 type evolving into a polytropic sequence like MLV002 or MLV006 (Figure 3), Similarly it seems implausible that MLV001, which shares with 9C a deletion encoding 15 amino acids of matrix (MA), presumably precluding virus replication, could evolve from cfs1. In the absence of evidence for replication competent MLV in the samples reported by Lo and colleagues, we believe that the finding of a population of gag sequences in the reagents, as well as the coincidence of a virtually identical replication incompetent MLV in our study and that of Lo and colleagues, must call into question the biological provenance of these sequences and therefore any conclusions drawn [5] concerning their relationship to CFS.

We found that contamination can be sporadic and so not immediately apparent. Although with one manufacturer's kit virtually all water controls gave amplification (Qiagen QuantiTect Virus Kit), the contamination described in this study with IPT reagents only became apparent once a large number of known negative samples (water) were amplified. This contamination of the Taq reagents with murine DNA sequences may be due to the enzyme mix itself and a consequence of the hot start mechanism. IPT has a mouse monoclonal antibody blocking the active site which is heat denatured for activation. This could contain murine DNA, potentially in variable amounts in different lots.

We do not believe that our observations serve to indicate that XMRV/pMLV sequences detected *ex vivo* in human materials inevitably will have come from the amplification reagents. Nor for that matter can we explain how in some studies there have been very significant differences between the detection rates in cases and comparator groups.

We suggest that contamination may complicate studies of XMRV in multiple ways depending on the source of the adventitious nucleic acid. Presence of material (RNA or DNA) from 22Rv1 cells would result in sequences identical or nearly identical to XMRV. Alternatively, mouse DNA could be the problem in other studies, when a variety of endogenous MLV will be amplified resulting in a range of related sequences as seen in this study. In particular we caution that the reagents used to detect MLV-like sequences may themselves have been contaminated with murine DNA. Although we do not suggest that this is the primary source of the XMRV/pMLV sequences observed in previous studies, it could well represent a confounding factor and note that the original detection of XMRV gag sequences by Urisman and colleagues used Invitrogen Taq and Lo and colleagues used IPT reagents. Finally we believe we would be wise to remember the classical warning “Caveat Emptor”.

## References

[1] Urisman A, Molinaro RJ, Fischer N, Plummer SJ, Casey G (2006). Identification of a novel Gammaretrovirus in prostate tumors of patients homozygous for R462Q RNASEL variant.. PLoS Pathog.

[2] Qiu X, Swanson P, Luk KC, Tu B, Villinger F (2010). Characterization of antibodies elicited by XMRV infection and development of immunoassays useful for epidemiologic studies.. Retrovirology.

[3] Lombardi VC, Ruscetti FW, Das Gupta J, Pfost MA, Hagen KS (2009). Detection of an infectious retrovirus, XMRV, in blood cells of patients with chronic fatigue syndrome.. Science.

[4] Mikovits JA, Lombardi VC, Pfost MA, Hagen KS, Ruscetti FW (2010). Detection of an infectious retrovirus, XMRV, in blood cells of patients with chronic fatigue syndrome.. Virulence.

[5] Lo SC, Pripuzova N, Li B, Komaroff AL, Hung GC (2010). Detection of MLV-related virus gene sequences in blood of patients with chronic fatigue syndrome and healthy blood donors.. Proc Natl Acad Sci U S A.

[6] Hohn O, Strohschein K, Brandt AU, Seeher S, Klein S (2011). No Evidence for XMRV in German CFS and MS Patients with Fatigue Despite the Ability of the Virus to Infect Human Blood Cells In Vitro.. PLoS One.

[7] Erlwein O, Kaye S, McClure MO, Weber J, Wills G (2010). Failure to detect the novel retrovirus XMRV in chronic fatigue syndrome.. PLoS One.

[8] Groom HC, Boucherit VC, Makinson K, Randal E, Baptista S (2010). Absence of xenotropic murine leukaemia virus-related virus in UK patients with chronic fatigue syndrome.. Retrovirology.

[9] Hong P, Li J, Li Y (2010). Failure to detect Xenotropic murine leukaemia virus-related virus in Chinese patients with chronic fatigue syndrome.. Virol J.

[10] van Kuppeveld FJ, de Jong AS, Lanke KH, Verhaegh GW, Melchers WJ (2010). Prevalence of xenotropic murine leukaemia virus-related virus in patients with chronic fatigue syndrome in the Netherlands: retrospective analysis of samples from an established cohort.. BMJ.

[11] Verhaegh GW, de Jong AS, Smit FP, Jannink SA, Melchers WJ (2010). Prevalence of human xenotropic murine leukemia virus-related gammaretrovirus (XMRV) in dutch prostate cancer patients.. Prostate.

[12] Schlaberg R, Choe DJ, Brown KR, Thaker HM, Singh IR (2009). XMRV is present in malignant prostatic epithelium and is associated with prostate cancer, especially high-grade tumors.. Proc Natl Acad Sci U S A.

[13] Hohn O, Krause H, Barbarotto P, Niederstadt L, Beimforde N (2009). Lack of evidence for xenotropic murine leukemia virus-related virus(XMRV) in German prostate cancer patients.. Retrovirology.

[14] Fischer N, Hellwinkel O, Schulz C, Chun FK, Huland H (2008). Prevalence of human gammaretrovirus XMRV in sporadic prostate cancer.. J Clin Virol.

[15] Arnold RS, Makarova NV, Osunkoya AO, Suppiah S, Scott TA (2010). XMRV infection in patients with prostate cancer: novel serologic assay and correlation with PCR and FISH.. Urology.

[16] Mikovits JA, Huang Y, Pfost MA, Lombardi VC, Bertolette DC (2010). Distribution of xenotropic murine leukemia virus-related virus (XMRV) infection in chronic fatigue syndrome and prostate cancer.. AIDS Rev.

[17] McCormick AL, Brown RH, Cudkowicz ME, Al-Chalabi A, Garson JA (2008). Quantification of reverse transcriptase in ALS and elimination of a novel retroviral candidate.. Neurology.

[18] Ribet D, Harper F, Dupressoir A, Dewannieux M, Pierron G (2008). An infectious progenitor for the murine IAP retrotransposon: emergence of an intracellular genetic parasite from an ancient retrovirus.. Genome Res.

[19] Jern P, Stoye JP, Coffin JM (2007). Role of APOBEC3 in genetic diversity among endogenous murine leukemia viruses.. PLoS Genet.

[20] Switzer WM, Jia H, Hohn O, Zheng H, Tang S (2010). Absence of evidence of xenotropic murine leukemia virus-related virus infection in persons with chronic fatigue syndrome and healthy controls in the United States.. Retrovirology.

[21] Robinson MJ, Erlwein OW, Kaye S, Weber J, Cingoz O (2010). Mouse DNA contamination in human tissue tested for XMRV.. Retroviology.

[22] Henrich TJ, Li JZ, Felsenstein D, Kotton CN, Plenge RM (2010). Xenotropic murine leukemia virus-related virus prevalence in patients with chronic fatigue syndrome or chronic immunomodulatory conditions.. J Infect Dis.

[23] Sato E, Furuta RA, Miyazawa T (2010). An endogenous murine leukemia viral genome contaminant in a commercial RT-PCR Kit is amplified using standard primers for XMRV.. Retrovirology.

[24] Oakes B, Tai AK, Cingöz O, Henefield MH, Levine S (2010). Contamination of human DNA samples with mouse DNA can lead to false detection of XMRV-like sequences.. Retrovirology.

[25] Hué S, Gray ER, Gall A, Katzourakis A, Tan CP (2010). Disease-associated XMRV sequences are consistent with laboratory contamination.. Retrovirology.

